# The past and present Earth-Moon system: the speed of light stays steady as tides evolve

**DOI:** 10.1186/s13535-014-0002-5

**Published:** 2014-04-04

**Authors:** James G Williams, Slava G Turyshev, Dale H Boggs

**Affiliations:** Jet Propulsion Laboratory, California Institute of Technology, Pasadena, CA 91109 USA

**Keywords:** Moon, Tides, Lunar tidal acceleration, Lunar orbit evolution, Speed of light, Lunar laser ranging, Earth rotation, 06.30.Gv Measurements common to several branches of physics and astronomy; Velocity, 91.10.Tq Solid earth physics; Geodesy and gravity; Earth tides, 96.12.De Solar system; Solid surface planets; Orbital and rotational dynamics

## Abstract

Tides induce a semimajor axis rate of +38.08 ± 0.19 mm/yr, corresponding to an acceleration of the Moon’s orbital mean longitude of −25.82 ± 0.13 "/cent^2^, as determined by the analysis of 43 yr of Lunar Laser Ranging (LLR) data. The LLR result is consistent with analyses made with different data spans, different analysis techniques, analysis of optical observations, and independent knowledge of tides. Plate motions change ocean shapes, and geological evidence and model calculations indicate lower rates of tidal evolution for extended past intervals. Earth rotation has long-term slowing due to tidal dissipation, but it also experiences variations for times up to about 10^5^ yr due to changes in the moment of inertia. An analysis of LLR data also tests for any rate of change in either the speed of light *c* or apparent mean distance. The result is (−2.8 ± 3.4)×10^–12^ /yr for either scale rate or –(d*c*/d*t*)/*c*, or equivalently −1.0 ± 1.3 mm/yr for apparent distance rate. The lunar range does not reveal any change in the speed of light.

## Background

The gravitational attraction of the Moon at the Earth causes a tidal distortion of the oceans and solid Earth. The tidal bulges are not quite aligned with the direction to the Moon. The orientation of the bulges leads the direction to the Moon, because a delayed response is carried forward by Earth rotation. There results a forward acceleration on the Moon and a deceleration of the Earth’s spin; energy and angular momentum are transferred from the Earth to the lunar orbit. The Moon’s mean distance increases and its orbit period increases. Analysis of accurate laser measurements of the range between observatories on the Earth and retroreflectors on the Moon determines these orbit changes with 1/2% accuracy. The “Calculation of lunar orbit anomaly” paper by Riofrio [1] claims that the present 38.1 mm/yr Lunar Laser Ranging (LLR) value for the tidal recession of the Moon is 9–10 mm/yr too large because a decreasing speed of light causes an apparent increase in distance. To support this idea, evidence from geology, Earth rotation, and ocean model calculations are cited. We discuss the tide-related geological and geophysical evidence. We outline the LLR sensitivity to both lunar tidal recession rate and increasing orbit period (0.352 ms/yr), and compare the recent values with older determinations. Analysis of LLR data also tests whether the speed of light is constant.

## Results and discussion

### Lunar tidal acceleration and recession rate

The transfer of energy and angular momentum from the rotation of the Earth to the orbit of the Moon causes the length of day, the lunar distance, and the lunar orbit period to increase. By deducing this mechanism, tidal recession was predicted theoretically by George Darwin in the late 19th century prior to its detection [2,3]. By convention, the tidal increase in the lunar orbit period is presented as a tidal decrease in mean motion that is equal to a negative tidal acceleration in orbital longitude. Tidal acceleration was detected and presented by Spencer Jones [4] and Clemence [5] in the last century from the analysis of optical observations of the Moon, Sun, and planets. According to Kepler’s third law, a negative acceleration in orbital mean longitude (mean longitude = mean anomaly + argument of perigee + node, and its derivative is mean motion) corresponds to a linear increase in semimajor axis. Tidal acceleration is frequently denoted by d*n*/d*t* and semimajor axis rate by d*a*/d*t*. The third-law connection 2*a* d*n*/d*t* + 3*n* d*a*/d*t* = 0 gives a first approximation. Some selected values follow:

1. Morrison and Ward [6] found a lunar tidal acceleration in longitude of −26 ± 2 seconds of arc/century^2^ (“/cent^2^) from the analysis of optical observations. Analysis of timings of transits of Mercury across the Sun from 1677–1973 allowed the changing angular rotation of the Earth to be determined separately from the lunar orbital tidal acceleration. Before accurate clocks became available in the middle of the last century, Earth rotation was a “clock” for celestial observations. The deceleration and other variations in Earth rotation needed to be determined with respect to a uniform physical time scale in order to determine the lunar tidal acceleration with respect to that time scale. The orbit of Mercury provided the uniform time scale.
2. Dickey et al. [7] determined a lunar tidal acceleration in longitude of −25.88 ± 0.5 “/cent^2^ and a semimajor axis rate of +38.2 ± 0.7 mm/yr from analysis of 24 yr of Lunar Laser Ranging (LLR) data. The lunar orbit and orientation were generated by numerical integration.
3. Chapront et al. [8] found a tidal acceleration of −25.858 “/cent^2^ from analysis of LLR data. They used series representations for lunar orbit and orientation.
4. Williams et al. [9,10] obtained a tidal acceleration of −25.85 “/cent^2^ and a semimajor axis rate of +38.14 mm/yr for the DE421 lunar ephemeris, which was derived from analysis of 38 yr of LLR data. DE421 was integrated numerically.
5. Williams et al. [11] determine a tidal acceleration of −25.82 ± 0.13 “/cent^2^ and a semimajor axis rate of +38.08 ± 0.19 mm/yr for the recent DE430 lunar ephemeris, which is derived from analysis of 43 yr of LLR data (18,548 ranges from March 1970 to December 2012).

The agreement between the Morrison and Ward [6] result and the LLR analyses [7–11] demonstrates that optical observations, mainly occultation timings, and laser ranges detect the same tidal acceleration. The tidal acceleration value derived by Chapront et al. [8] shows that an independent approach and software yields compatible LLR results. The JPL results [7,9–11] demonstrate that with increasing data span and improving uncertainty the tidal acceleration determination is stable.

Ordered by decreasing contribution, the M2, O1, and N2 tides determine most of the tidal acceleration. But for eccentricity rate the order is N2, Moon tides, Q1, and M2, with the second and last being negative. The tide model for DE421 adjusted one time-delay dissipation parameter for semidiurnal tides, one for diurnal tides, and one for Moon tides. These parameters fit the tidal acceleration well, but were less successful with the eccentricity rate [10]. The model for tidal perturbations from tides on the Earth was improved prior to DE430 [11] to allow for time-delay shifts across the diurnal and semidiurnal bands. The DE430 tidal eccentricity rate is 1.4×10^–11^/yr, with 1.8×10^–11^/yr coming from tides on the Earth and −0.4×10^–11^/yr from solid-body tides on the Moon. This is an improvement over DE421 (0.9×10^–11^/yr), but when an analytical eccentricity rate solution parameter is added to post-DE430 LLR analyses, then an additional rate is found and the total eccentricity rate becomes (1.9 ± 0.2)×10^–11^/yr. The extra eccentricity rate, in addition to the rate from our tidal model, implies that further dissipation-related modeling improvements are possible.

There is further evidence supporting the modern determinations of lunar tidal acceleration and recession. Tides on the Earth have been studied with satellites. Altimetry measures ocean tide heights, and tidal attraction is determined from perturbations on satellite orbits. These studies separate the tides into different periodic components. For example, the largest semidiurnal tide is the M2 tide with a period of 12.42 hr, and the largest diurnal tide is the O1 tide with a 25.82-hr period. There are also slow zonal tides with periods of one month and one-half month. Tides raised by the Sun are about half the size of tides raised by the Moon. Lunar tidal acceleration is mainly caused by the gravity from Moon-raised tides on the Earth acting back on the Moon. Lunar tidal acceleration, computed from the satellite-determined tidal components presented by Lyard et al. [12] and Ray [13], compared favorably with the tidal acceleration of the LLR-derived DE421 lunar orbit [9,10]. Williams et al. [10] found a difference of less than 1% between the two values. The DE430 tidal acceleration [11] also agrees within 1%.

The lunar tidal anomaly paper [1] focuses its discussion on the semimajor axis rate *da*/*dt*, rather than the acceleration in orbital mean longitude. The LLR data analyses are more sensitive to the acceleration in mean anomaly, a near proxy for the acceleration in mean longitude^a^, than to the recession rate because the lunar orbit is eccentric [14]. Because there is a monthly variation of the radius of the lunar orbit, any perturbation of mean anomaly will cause a perturbation in that radius. For example, the tidal acceleration causes a −2.4 m/yr^2^ *t*^2^ perturbation in the product of semimajor axis *a* and mean longitude perturbation, and an approximate +0.038 m/yr *t* – 0.13 m/yr^2^ *t*^2^ sin(mean anomaly) perturbation in radius, where the time *t* in years is zero at the epoch when the perturbation starts to accumulate. The oscillating *t*^2^ term in radius is much stronger than the linear *t* term from the semimajor axis for times of years to decades. By only considering the increasing semimajor axis, [1] incorrectly assumed that LLR data analysis would mistake any constant rate in radius for a tidal perturbation. The perturbation in orbital radius from the DE430 tidal eccentricity rate is approximately −0.005 m/yr *t* cos(mean anomaly), distinct from either a linear increase or a *t*^2^ sin(mean anomaly). The foregoing expressions are for illustration; the LLR programs use an integrated orbit.

### The moon’s evolving orbit and the earth’s decelerating spin

The lunar anomaly paper [1] offered three types of evidence in support of a slower semimajor axis rate: geological evidence from a rhythmite, ocean model calculations of tides, and nontidal acceleration of the Earth’s rotation.

#### Rhythmites

Tidal rhythmites preserve geological layering from ancient tides, and they may allow the past evolution of the Moon to be unraveled. Modulation of the layers may permit the number of days per month, days per year, or months per year to be determined after identifying the cause of each periodicity. The lunar anomaly paper [1] selected the 310 million year old Mansfield sediment to derive a 2.9 ± 0.6 cm/yr average lunar recession rate over the 310 million year interval. A review of tidal rhythmites and related structures is presented by Coughenur et al. [15]. This review gives a 2.17 ± 0.31 cm/yr average recession rate for the 620 million year old Reynella Siltstone, a member of the Elatina Formation. We accept the idea that the past rate of lunar recession was lower than the present value for extended spans of time, but we do not accept the practice of using a past rate to replace or assess the accuracy of the current rate.

Most of the tidal dissipation that causes the recession of the Moon occurs in the oceans. The pattern of each tidal component is complicated; see Coughenur et al. [15] and Poliakow [16] for examples. Each tidal component has an individual period, and for each the pattern of local tide heights and phases depends on location. The combination of tidal components also depends on location, a complication for the analysis of rhythmites. The pattern for each tidal component can be combined into a global series of spherical harmonic functions. In a global sense, energy dissipation causes each tidal component to shift orientation with respect to the Moon’s attraction. This phase-shifted part of each tidal component causes lunar tidal acceleration, semimajor axis rate, and eccentricity rate. Plate motion changes the shapes and locations of the oceans, and this causes substantial variations in the tidal acceleration over ~10^8^ yr time scales [16]. Although the tidal acceleration varies over these long time scales, the Moon continues to move outward.

#### Tide models

Poliakow [16] computed the past evolution of one tidal component, M2, the largest semidiurnal component. The M2 tidal component contributes ~80% of the current total tidal acceleration. Although the M2 tide does not cause the total recession rate, the lunar anomaly paper [1] cites the M2 calculation of a current +29 mm/yr contribution [16] as though the M2 rate was the total recession rate. For comparison, the DE430 lunar ephemeris has a 31 mm/yr recession rate caused by the M2 component. Other DE430 rates are +33.5 mm/yr from all semidiurnal tides, +5.1 mm/yr from all diurnal tides, and −0.5 mm/yr from zonal tides on the Earth and tides on the Moon [11]. Although [1] cites Poliakow [16] for support for a 29 mm/yr recession rate, it contradicts his calculations for large variation in the past M2-caused rate by saying “For the Moon’s recession to vary so greatly, tidal heights would have to increase enormously over time.” In addition to contradicting the cited paper, this statement seems to confuse the roles of local tide heights that influence rhythmites and phase-shifted components of global tides that cause the lunar recession rate. Any claim of steady tide heights based on rhythmites must be viewed with skepticism.

Over 10^8^ yr time scales, the oceans changed shape due to plate motion, which affected tides. Over even longer time scales, the more rapidly spinning Earth of the past shifted the tidal frequencies with respect to the resonant (normal mode) frequencies of the oceans. Bills and Ray [17] considered several models of changing ancient tides including models of Webb [18] and Hansen [19]. Those studies and [16] found that past tidal dissipation varied by large amounts. Although Bills and Ray concluded that present-day tidal dissipation was more effective^b^ than in the past, they considered the reason to be understood. Despite the understanding demonstrated by [16–19], [1] implies that the Bills and Ray work considered differences between past and present tidal recession rates to be an anomaly. Tides evolve due to plate motion and slowing spin rate.

#### Nontidal acceleration of Earth rotation

The increasing orbital angular momentum of the Moon’s evolving orbit is supplied by a decreasing terrestrial spin angular momentum. Determinations of the deceleration of Earth rotation by Stephenson and Morrison [20] yield about 3/4 of the deceleration expected from lunar tidal acceleration^c^. The difference is called nontidal acceleration. The cause of the nontidal acceleration of Earth rotation was explained three decades ago by Yoder et al. [21]. They found that the Earth’s oblate shape and moment of inertia *C* are decreasing. Since spin angular momentum *Cω* is the product of the Earth’s moment and spin rate *ω*, a negative d*C*/d*t* causes a positive contribution to d*ω*/d*t* since d*ω*/d*t* = [*T* – *ω* d*C*/d*t* ]/*C*, where the tidal torque *T* is negative. From the analysis of satellite tracking data, the Earth’s d*J*_2_/d*t* was determined to be negative by [21], where *J*_2_ is the degree-2 coefficient of the gravitational potential that describes the oblateness of the Earth’s gravity field. Since d*C*/d*t* = (2/3) *MR*^2^ d*J*_2_/d*t*, where *M* is the mass and *R* is the equatorial radius, the change in moment *C* is established. The nontidal acceleration of rotation from (d*ω*/d*t*)_NT_ = −*ω* (d*C*/d*t*)/*C* agrees with the historical nontidal acceleration from [20].

The Earth’s moment of inertia and rotation are affected by short- and long-term effects. Short-term effects can include ocean and atmospheric mass redistribution, elastic response to changing loads, and a tide that changes the moment of inertia with an 18.6-yr period. A recent Cheng et al. [22] analysis finds a negative *J*_2_ rate from 1976 to ~1995 that agrees with Yoder et al. [21], but the rate subsequently decreases, possibly due to (short-term) modern global warming and the resulting redistribution of water mass. Stephenson and Morrison [20] analyzed Earth rotation over 2700 yr, so long-term changes apply. The initial determination of d*J*_2_/d*t* led [21] to an explanation for the nontidal acceleration of the Earth’s rotation. The negative d*C*/d*t* was interpreted to be from viscous rebound of the Earth following deglaciation near the end of the last ice age ~10^4^ yr ago. The weight of glaciers depressed the surface of the Earth under the polar ice during the ice ages, and after melting removed that weight the Earth’s surface started rebounding upward. Viscous rebound is slow lasting thousands of years and continuing today. The slow shape change from viscous rebound causes a nontidal acceleration of Earth rotation with a sign opposite to that of tidal deceleration. Over the longer ~10^5^ yr time scale of ice age cycles, the nontidal acceleration changes sign and is not truly secular. The past decrease of *J*_2_ has been detected, and the directly linked nontidal acceleration of rotation applies to historical data.

Under Possible explanations, the lunar anomaly paper [1] attempts to mention and discount the viscous rebound explanation, but the statements there are confusing. The viscous rebound of the Earth has an exponential relaxation time of several thousand years; large-scale deglaciation is only required near the end of the last (quaternary) ice age ~10–15 thousand years ago, so extensive deglaciation is not required for historical times. Nontidal acceleration of Earth rotation does not change tidal friction, but it complicates any use of the deceleration of Earth rotation to infer lunar tidal recession, which [1] attempts.

### Testing whether the speed of light decreases

The lunar anomaly paper [1] proposes that the speed of light *c* is slowing with time. Although a slowing speed of light would cause an increase in the apparent lunar distance, it would not change the tidal acceleration in orbital longitude, already conflicting with the observational results given earlier. Still, an apparent nontidal increase in distance or scale is a testable prediction. LLR data were analyzed to seek any rate of change of the round-trip time of the laser pulse, the “range,” that was distinct from lunar tidal acceleration and recession [23]. Apart from tidal recession, [23] found a limit for the absolute value of any anomalous distance rate of <3.5 mm/yr, a limit that converts to |scale rate| = |(d*c*/d*t*)/*c*| <0.9×10^–11^ /yr. This limit is smaller than the prediction in [1] of −2.4×10^–11^/yr for (d*c*/d*t*)/*c*, or +9 mm/yr in apparent distance. Although [1] cited the LLR paper on relativity [23], it did not mention this result that contradicts the d*c*/d*t* prediction. That earlier solution [23] is updated here: we fit 18,696 laser ranges between March 1970 and April 2013; in addition to scale rate, LLR solution parameters include diurnal and semidiurnal tidal acceleration parameters, tidal dissipation in the Moon, an eccentricity rate in addition to that caused by our tidal model, parameters for lunar orbit (including mean distance) and orientation, locations of ranging stations and retroreflector arrays, and other standard LLR solution parameters [11]. Folkner et al. [24] detail the formulation for lunar orbit and orientation. For scale rate, or –(*dc*/*dt*)/*c*, we obtain (−2.8 ± 3.4)×10^–12^ /yr, or −1.0 ± 1.3 mm/yr in apparent distance. This test result is much smaller than the d*c*/d*t* prediction of [1]. The correlations between the diurnal and semidiurnal tidal acceleration parameters and the scale rate parameter are small, –0.03 and +0.03, respectively, supporting the earlier assertion that a *t*^2^ sin(mean anomaly) perturbation of orbital radius is distinct from a linear increase. The correlation between eccentricity rate and scale rate is −0.14, and its correlations with the two tidal acceleration parameters are −0.05 and +0.19, respectively. There is a good separation of parameters.

The age of the expanding universe is 1.38×10^10^ yr. The scale rate computed from the inverse age is 0.72×10^–10^ /yr; this is the Hubble constant expressed with different units than the usual km/s/Mpc. The lunar semimajor axis rate gives (d*a*/d*t*)/*a* = 0.99×10^–10^ /yr, where *a* = 384,399 km. This similarity of numbers led Van Flandern [25,26], before Riofrio [1], to attempt to link the lunar recession rate to cosmology. He proposed that tidal acceleration would be different for atomic and dynamical time scales, and the time scale difference would be caused by a decreasing gravitational constant *G* that was linked to the Hubble constant. Modern results do not support either a difference in time scales, e.g., the agreement of [6] with [7–11], or a changing *G* [23,27,28]. The similar values of (d*a*/d*t*)/*a* and the Hubble constant are due to multi-billion year ages for the Earth-Moon system and the universe. The Earth, Moon, and solar system are ~4.55×10^9^ yr old. The lunar (d*a*/d*t*)/*a* must be smaller than the inverse age of the Earth-Moon system since the tidal recession rate was faster when the Moon was close to the Earth. The solar system age is about 1/3 of the age of the universe, so the similarity of the two rates does not require an unusual explanation. The lunar range provides no observational evidence for a slowing of the speed of light, or any other connection between the apparent lunar recession rate and the age of the universe.

## Conclusions

Present day lunar tidal acceleration values are consistent between optical occultation timings and laser range techniques, different analysis approaches and programs, and prediction from satellite measurement and modeling of tides. Lunar laser ranges are sensitive to tidal acceleration in mean anomaly as well as increasing semimajor axis. Concerning the evidence for a lower recession rate offered by Riofrio’s lunar anomaly paper [1]: (1) the geological record does not establish the present rate, (2) modeling of one tidal component does not give the total lunar tidal acceleration and recession, and (3) there is a geophysical origin for the nontidal acceleration of Earth rotation. The cosmological suggestion that the speed of light is slowing has been tested and does not match the prediction. The alleged lunar orbit anomaly does not exist and cosmological inferences are not warranted. Tides evolve, but the speed of light remains steady.

## Endnotes

^a^The tidal acceleration in mean anomaly and mean longitude are nearly the same. The tidal accelerations of the argument of perigee and node are smaller by two orders of magnitude. The practice of giving the acceleration in mean longitude is a convention from the days of optical observations of angles.

^b^The computation of dissipation-induced orbit and rotation changes from each global tidal component involves a ratio of Love number *k*_2_ divided by a specific dissipation *Q* for the component. Large values of *k*_2_/*Q* are referred to as more effective here. For a global representation of tides, although most of the phase-shifted tide comes from the oceans, the in-phase part comes mainly from solid-body tides. For the global M2 tide, LLR analysis has *k*_2_/*Q* ≈ 0.32/13 with a phase shift of 4.5˚.

^c^The orbital tidal acceleration is mainly sensitive to dissipation from tides raised by the Moon on the Earth, with a 1% effect from tides raised by the Earth on the Moon [11,29]. However, the tidal deceleration of the Earth’s rotation is sensitive to tides raised on the Earth by both Moon and Sun. Also, the K1 tidal dissipation affects rotation, but not orbit. Consequently, although the larger part of the tidal deceleration of rotation is from tidal components that also affect the orbit, a tidal model is required for part of the rotation computation. This can be as simple as a constant phase shift, or as sophisticated as an ocean model such as [12,13].

## Abbreviation

JPL: Jet propulsion laboratory
LLR: Lunar laser ranging
